# Aetiology of diarrhoea in children aged zero to nine years in low- and middle-income countries: A systematic review

**DOI:** 10.7189/jogh.14.04168

**Published:** 2024-11-01

**Authors:** Sinjini Das, Raghavee Neupane, Jennifer Beard, Hiwote Solomon, Monalisa Das, Neil Errickson, Jon L Simon, Yasir B Nisar, William B MacLeod, Davidson H Hamer

**Affiliations:** 1 Department of Global Health, Boston University School of Public Health, Boston, Massachusetts, USA; 2 Department of Maternal, Newborn, Child and Adolescent Health and Ageing, World Health Organization, Geneva, Switzerland; 3 Section of Infectious Diseases, Department of Medicine, Boston University Chobanian and Avedisian School of Medicine, Boston, Massachusetts, USA; 4 Centre on Emerging Infectious Diseases, Boston University, Boston, Massachusetts, USA; 5 Friedman School of Nutrition Science and Policy, Tufts University, Boston, Massachusetts, USA

## Abstract

**Background:**

While diarrhoeal disease remains a leading cause of death in children aged <5 years in low- and middle-income countries (LMICs), it also poses significant health risks for older children, underscoring the importance of our study focusing on children aged <10 years. In this systematic review, we assessed common diarrhoea aetiologies in children aged <10 years in LMICs.

**Methods:**

We identified relevant articles in PubMed, Embase, and Web of Science using pre-defined search criteria. We included case series and case-control studies of children aged <10 years with non-bloody, bloody, acute, persistent, and chronic diarrhoea. Articles that evaluated two or more diarrhoea pathogens in LMICs conducted between 1 January 1990 and 31 July 2020 were eligible for inclusion. We stratified combined data from case series and case-control studies by age and World Health Organization (WHO) regions.

**Results:**

76 studies published between 1990–2020 were eligible for inclusion. Among these, eight were case-control studies. 56 papers focused only on children aged <5 years, while 20 also included children aged ≥5 years. The most common viral pathogens among <5 years old children were rotavirus, norovirus, adenovirus, and astrovirus. Bacterial pathogens included *Escherichia coli*, *Salmonella enterica*, *Shigella* species, and *Campylobacter* species, while parasitic pathogens included *Cryptosporidium*, *Giardia*, and *Entamoeba* species. Rotavirus was the most common viral pathogen among children across all age groups and every WHO region. *Escherichia coli* was prevalent in all age groups and was responsible for most diarrhoea cases in the African Region. Among parasitic pathogens, *Entamoeba* species and *Giardia* were prevalent in children aged three to five years, with the former a major cause of diarrhoea in the Eastern Mediterranean Region. Similarly, in children aged six to 10 years, bacterial pathogens, including *Escherichia coli, Salmonella*, and *Shigella*, suggest a continued significance of these pathogens beyond the age of five. Common viral pathogens for this group were rotavirus, norovirus, and sapovirus, although the number of studies for this age group is limited.

**Conclusions:**

*Escherichia coli*, rotavirus, and *Entamoeba* species were the most common pathogens responsible for diarrhoea in children aged <5 years in LMICs. Future research should focus on characterising the pathogens responsible for causing diarrhoea in children aged six to 10 years stratified by geographic area of residence, i.e. WHO region and urban vs rural. Case-control or cohort studies covering a full 12-month period to account for seasonality are needed for a more accurate picture of diarrhoea aetiology among children.

**Registration:**

PROSPERO (CRD42020204005).

Diarrhoeal diseases are among the leading causes of death in children aged <5 years in low- and middle-income countries (LMICs) [1]. Diarrhoea was estimated to account for 9% of deaths in children aged <5 years worldwide in 2013 (the most recent data available) [1]. There were about 444 000 deaths of children under 5 years of age in 2022 and an estimated half a million when including all children <10 years old, predominantly in LMICs, highlighting the persistent challenge of childhood deaths due to diarrhoea. Despite the availability of effective interventions, there has been a slight reduction in diarrhoeal disease episodes and mortality in recent years [2,3].

The World Health Organization (WHO) defines diarrhoea as passing three or more loose stools (or more frequent passage than is normal for the individual) within one day [4]. Episodes of diarrhoea lasting less than 14 days are defined as acute, and those lasting for more than 14 days as persistent [1].

Childhood diarrhoea can be caused by a broad range of viral, bacterial, and parasitic organisms [5]. The most common pathogens include rotavirus, toxigenic strains of *Escherichia coli*, *Shigella*, *Vibrio cholerae*, and *Salmonella* species. Some studies have reviewed the aetiology of diarrhoea in children aged <5 years, including well-designed, multi-country, case-control studies like the Global Enteric Multicentre Study (GEMS) [6]. Additionally, a few studies have evaluated the age-specific pathogenicity of diarrhoeal disease in children aged six to 10 years in LMICs [6]. However, research specifically addressing the causes of diarrhoea in this older age group within LMICs is limited [7].

We conducted a systematic review of research assessing the causes of diarrhoea, including acute, persistent, and chronic cases, in children aged <10 years in LMICs and examined regional differences in diarrhoea aetiology. Our overarching goal was to address the following question: what are age-specific patterns and geographic differences for diarrhoeal aetiologies in children aged <10 years in LMICs? We also extracted data on the number and proportion of children infected with diarrhoeal disease, broken down by organism, age, and WHO regions, from each article to provide a comprehensive summary of diarrhoea causes across different age groups and regions.

Our synthesis of this body of research can inform public health strategies, interventions, and policies to effectively prevent and manage diarrhoeal diseases, particularly in LMICs where the burden is highest [7].

## METHODS

We registered the systematic review protocol at PROSPERO (registration number CRD42020204005). We searched PubMed, Embase, and Web of Science using different combinations of keywords, medical subject headings terms, and headings: diarrhoea, aetiology, children, infants, pathogen, incidence, mortality, cause of death, causation, acute, persistent, bloody, newborn, child, causation, preschool, risk factors, and gastroenteritis.

The following types of studies, conducted in countries defined by the World Bank as low or middle-income, were eligible for inclusion: observational, cohort, surveys, case-control, database analyses, or randomised controlled trials published between 1 January 1990 and 31 July 2020 [8]. We only included studies of children aged <10 years that tested for more than one pathogen, provided a clearly defined laboratory methodology, and stratified findings by age (such as 0–1 year, 1–2 years, 2–3 years, 4–5 years, and 5–9 years). Given the numerous variations among studies, such as diverse sampling strategies and microbiology methods, we opted for a systematic review instead of a meta-analysis.

We conducted the search and systematic data extraction from July to October 2020. Two researchers, MD and NE, collaborated to identify potentially relevant documents through a comprehensive screening process. Any discrepancies were promptly addressed by a third researcher, DHH, who ultimately determined the paper’s inclusion based on established criteria. We examined the bibliographies of review papers to identify potentially relevant resources. The remaining potentially eligible documents were screened to determine whether they matched the inclusion criteria.

We extracted the number and proportion of children infected with diarrhoeal disease broken down by organism, age, and WHO regions – African Region (AFR), Region of the Americas (AMR), Eastern Mediterranean Region (EMR), European Region (EUR), South-East Asian Region (SEAR), and Western Pacific Region (WPR). We thoroughly examined diarrhoea caused by specific pathogens, detailing the average proportions for each year for the age group <10 years. We have also categorised the data by WHO region, distinguishing between viral, bacterial, and parasitic pathogens. We integrated data from case series and case-control studies to comprehensively summarise diarrhoea causes across age groups and WHO regions. Data from case series and case-control studies were combined to summarise the causes of diarrhoea by age group and WHO region.

The following toxigenic strains were pooled for analysis as toxigenic *Escherichia coli*: enterotoxigenic *Escherichia coli* (ETEC), enteroaggregative *Escherichia coli* (EAggEC), enterohemorrhagic *Escherichia coli* (EHEC), enteroinvasive *Escherichia coli* (EIEC), and enteropathogenic *Escherichia coli* (EPEC). *Giardia* and *Giardia lamblia* were combined and collectively referred to as *Giardia* species. Likewise, *Vibrio cholerae* and *Vibrio parahaemolyticus* were pooled as *Vibrio* species. Since most studies did not use specialised testing to differentiate *Entamoeba histolytica* from *Entamoeba dispar*, these were combined into one category, *Entamoeba* species (Table S1 in the **Online Supplementary Document**).

## RESULTS

**Figure 1:**
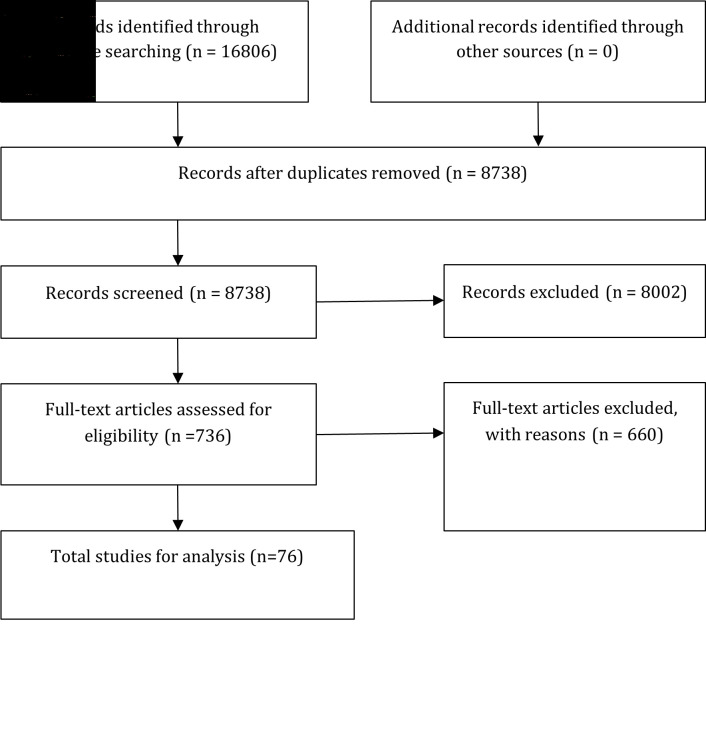
Article extraction protocol.

We identified 16 806 articles from our initial search. After removing duplicate articles, 8738 articles remained. After an initial screening of abstracts and titles, 736 publications were assessed for eligibility. An additional 660 articles were removed from the review because they did not meet the inclusion criteria. We analysed 76 studies (**Figure 1**).

### Diarrhoeal disease aetiology among children aged <5 years

Of the 76 studies reviewed, five included data only on children aged up to two years, and an additional 20 studies included children between three and five years old. 22 studies included bacteria, viruses, and parasites. The remaining 54 studies focused on either common viral pathogens such as rotavirus, norovirus, adenovirus, and astrovirus, bacterial pathogens including *Escherichia coli*, *Salmonella* species, *Shigella* species, and *Campylobacter* species, or protozoal parasites, in particular *Cryptosporidium*, *Giardia*, and *Entamoeba* species. Bacterial pathogens were the most assessed, followed by viruses and then parasites. We report results on six genera of bacterial, five viral, and three genera of parasitic pathogens. Only eight studies included healthy subjects (**Table 1**). A stratified breakdown of aetiology results by age is provided in **Table 2**. Analysis of diarrhoeal aetiology studies by pathogen and age group showed that *Escherichia coli* leads with 47 studies in the age group of 0–11 months, closely followed by rotavirus, which has 43 studies in the same group. *Shigella* also shows notable counts, particularly with 39 studies in the 0–11 and 24 studies in the 12–23 months category. Norovirus and adenovirus are also prominent among younger age groups, with 25 and 29 studies, respectively, in the age group of 0–11 months. In contrast, sapovirus has the lowest representation, lacking cases in age groups >12 months. Overall, the total number of studies across all age groups is 294 (Table S1 in the **Online Supplementary Document**).

**Table 1:** All studies with control groups (including cohort studies and case-control studies)

Study	Data collection years	Location (WHO region)	Pathogens evaluated	Study design	Criteria for selection of control
Hasan et al. [9]	1993	Mirzapur, Bangladesh (SEAR)	Rotavirus, *Escherichia coli*, *Shigella* species, *Campylobacter*, *Giardia*	Cohort	Asymptomatic controls (children without loose stools on the day of the specimen collection and three days before and after the collection).
Li et al. [10]	2011–23	China (SEAR)	Rotavirus, norovirus GII, *Shigella* species, adenovirus, norovirus GI, astrovirus, sapovirus, *Salmonella* species, ETEC, *Campylobacter jejuni*	Case-control	Controls were matched by sex, age group (0–5, 6–11, 12–23, 24–35, 36–47, and 48–59 mo), and location of residence.
Bodhidatta et al. [11]	2001–02	Western Thailand (SEAR)	*Campylobacter, Salmonella*, *Shigella*, *Plesiomonas*, *Aeromonas*, *Vibrio*, *Arcobacter butzleri*, *Escherichia coli* (ETEC, EIEC, EPEC, STEC), rotavirus, astrovirus, adenovirus, *Cryptosporidium*, *Giardia lamblia*	Case-Control Study	Asymptomatic controls (non-diarrhoea controls-no history of diarrhoea for the two weeks before enrolment).
Albert et al. [12]	1993–94	Dhaka, Bangladesh (SEAR)	Rotavirus, *Campylobacter jejuni*, ETEC, EPEC, *Aeromonaa* species, *Vibrio cholerae* O1, *Vibrio cholerae* O139, ETBF, *Clostridium difficile*, *Cryptosporidium parvum*, *Salmonella* species, *Plesiomonas shigelloides*, *Giardia lamblia*, *Entamoeba histolytica*, EAEC, DAEC	Case-control	Controls were healthy children (not taken antibiotics during the previous two weeks).
Nhampossa et al. [13]	2007–11	Manhiça, Mozambique (AFR)	*Giardia lamblia*, *Cryptosporidium, Entamoeba histolytica*, *Entamoeba dispar*, rotavirus, adenovirus, norovirus, sapovirus, astovirus, ST-ETEC, LT-ETEC, EAEC, EPEC typical, EPEC atypical, *Shigella*, *Salmonella non-typhi*, *Aeromonas*, *Campylobacter*	Prospective, age-stratified and matched (by age, gender, and geographical origin)	For each child with moderate-to-severe, one to three healthy control children (no diarrhoea in the previous seven days) were randomly selected from the neighbourhood.
Nguyen et al. [14]	2002	Hanoi, Vietnam (WPR)	Group A rotavirus, *Escherichia coli*, *Shigella* species, ETBF	Case-control	249 age-matched healthy controls were enrolled from daycare and health care centres in Hanoi, Vietnam. They had no diarrhoeal episodes for at least one month before collecting faecal samples.
Breurec et al. [15]	2011–13	Central African Republic (AFR)	*Shigella* species/EIEC, *Salmonella enterica*, diarrhoeagenic *Escherichia coli*, *Entamoeba histolytica*, *Cryptosporidium parvum/hominis*, *Giardia intestinalis*, astrovirus, rotavirus, adenovirus, norovirus	Case-control	Controls matched by age, sex, and neighbourhood of residence of each case were included. Cases were significantly more severely or moderately malnourished than controls. To be eligible, controls had to be in good general health, with no history of diarrhoea or antibiotic use during the seven days before sampling. HIV status was not systematically tested in controls, but if parents spontaneously declared a child to be seropositive, that child was not included.
Kotloff et al. [6]	2005–07	Mali, Gambia, Mozambique, Kenya, India, Bangladesh, Pakistan (AFR, SEAR)	Rotavirus, *Cryptosporidium*, ST-ETEC, *Shigella*, *Aeromonas*, *Vibrio cholerae* O1, *Campylobacter jejuni*	Three-year, prospective, age-stratified, matched case-control study of moderate-severe diarrhoea in children aged 0–59 mo residing in censused populations at four sites in Africa and three in Asia	The controls had to reside in the demographic surveillance system area. They were matched to the index case in terms of age, same sex, residence, and time. The controls did not have diarrhoea in the previous seven days.

AFR – African Region, DAEC – diffusely-adherent *Escherichia coli*, EAEC – enteroaggregative *Escherichia coli*, EIEC – enteroinvasive *Escherichia coli*, EPEC – enteropathogenic *Escherichia coli*, ETBF – enterotoxigenic *Bacteroides fragilis*, ETEC – enterotoxigenic *Escherichia coli*, LT-ETEC – heat-labile enterotoxigenic *Escherichia coli*, SEAR – South-East Asian Region, STEC – Shiga toxin-producing *Escherichia coli*, ST-ETEC – heat-stable enterotoxigenic *Escherichia coli*, WHO – World Health Organization, WPR – Western Pacific Region

**Table 2:** Studies with their respective number of cases and controls within case-control studies and cohort studies mentioned in this paper

	Age in years (PWD/PWOD)				
**Study and pathogens**	**0–1**	**1–2**	**2–3**	**3–4**	**4–5**
Hasan et al. [9]					
*Rotavirus*	47/4	42/6			
*Escherichia coli*	327/74	305/55			
*Shigella* species	44/4	88/6			
*Salmonella*	11/11	10/11			
*Campylobacter*	69/72	40/161			
*Giardia*	57/138	174/605			
Li et al. [10]*					
*Rotavirus*	93/1	71/0		2/0	
*Norovirus GII*	53/4	23/4		3/0	
*Adenovirus*	29/8	17/4		4/0	
*Shigella*	3/0	0/0		5/1	
Bodhidatta et al. [11]*					
*Rotavirus*	19/7	14/0		8/0	
*Adenovirus*	18/3	8/0		7/1	
*ETEC*	6/0	6/6		8/7	
*ETEC total*	84/97	78/65		47/41	
*EPEC*	12/19	13/8		8/4	
*EPEC total*	91/105	80/71		48/43	
*Shigella*	3/1	6/0		12/0	
*Salmonella*	6/6	4/9		5/6	
*Campylobacter*	21/27	21/24		9/8	
*Giardia*	5/10	27/25		14/15	
*Plesiomonas*	5/9	5/9		13/9	
*Rotavirus total*	97/109	85/78		50/0	
*Adenovirus total*	85/103	79/78		43/47	
*Giardia total*	85/103	86/78		43/47	
Albert et al. [12]					
*Rotavirus*	118/6	34/6	7/0	4/0	2/0
*Campylobacter jejuni*	84/54	31/31	11/10	10/6	6/2
*ETEC*	83/30	34/30	11/9	8/2	1/1
*EPEC*	76/32	16/9	5/2	2/1	0/0
*Aeromonas* species	46/19	25/9	11/7	15/3	2/1
*Shigella* species	28/6	26/9	11/7	7/2	3/0
*Vibrio cholerae O1*	16/1	7/0	6/0	7/0	1/0
*Vibrio cholerae O139*	8/2	7/0	9/0	8/0	2/0
*ETBF*	16/7	6/4	2/1	2/0	2/0
*EAEC*	54/39	18/15	3/3	2/0	0/0
*DAEC*	19/35	11/19	2/5	7/3	1/0
Nhampossa et al. [13]*					
*Giardia lamblia*	41/152	64/228		42/115	
*Cryptosporidium*	84/86	44/46		11/18	
*Entamoeba histolytica/dispar*	39/79	26/52		15/28	
*Rotavirus*	182/139	52/91		12/27	
*Adenovirus*	13/30	10/12		0/6	
*Norovirus*	19/38	10/25		4/12	
*Sapovirus*	7/24	3/11		0/3	
*Astrovirus*	7/11	6/10		1/4	
*ETEC*	32/77	46/48		11/19	
*EAEC*	150/273	60/92		21/35	
*EPEC*	49/87	36/48		27/17	
*Shigella*	6/1	18/2		20/0	
*Salmonella*	6/6	2/0		NA	
*Vibrio cholerae*	NA	4/1		9/0	
*Aeromonas*	5/0	1/0		0/1	
*Campylobacter*	24/0	9/14		0/2	
Nguyen et al. [14]					
*Group A rotavirus*	111/1	102/4	42/3	12/1	7/0
*EAEC*	35/2	24/5	6/7	2/3	1/1
*EIEC*	2/0	6/0	2/0	0/0	2/0
*EPEC*	15/1	18/5	3/2	2/2	1/1
*ETEC*	2/0	2/1	4/0	3/0	2/0
*Shigella flexneri*	0/0	3/0	1/0	3/0	0/0
*Shigella sonnei*	1/0	5/0	5/0	6/0	3/0
*Shigella boydii*	1/0	NA	NA	NA	NA
*ETBF*	12/0	16/1	5/1	5/2	5/0
Breurec et al. [15]					
*Rotavirus*	90/8	43/2			
*Norovirus*	18/9	11/5			
*Adenovirus*	11/9	6/6			
*Astrovirus*	25/8	7/7			
*Escherichia coli*	24/17	10/13			
*Shigella*	19/15	30/15			
*Salmonella*	¼	8/3			
*Campylobacter*	3/5	3/3			
*Giardia*	0/6	2/2			
*Entamoeba*	0/1	1/3			
*Cryptosporidium*	32/7	7/2			

DAEC – diffusely-adherent *Escherichia coli*, EAEC – enteroaggregative *Escherichia coli*, EIEC – enteroinvasive *Escherichia coli*, EPEC – enteropathogenic *Escherichia coli*, ETBF – enterotoxigenic *Bacteroides fragilis*, ETEC – enterotoxigenic *Escherichia coli*, NA – not available, PWD – people with diarrhoea, PWOD – people without diarrhoea

*The age group of 3–4 years presents the study’s results for children with age >24 months.

The majority of studies (n = 25) were conducted in the WHO AFR, followed by SEAR (n = 15), AMR (n = 11), WPR (n = 11), EMR (n = 10), and EUR (n = 3). Two studies included multiple regions (Data set S1 in the **Online Supplementary Document**).

### Viral aetiologies among children aged <5 years with diarrhoea

**Figure 2:**
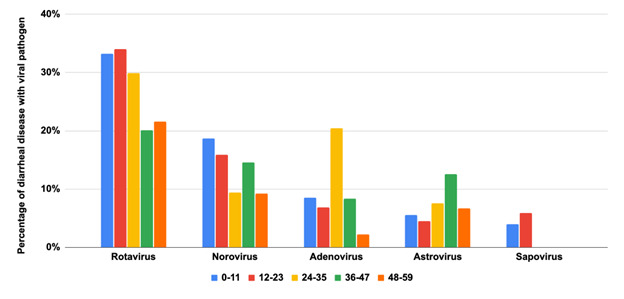
Viral pathogens are the cause of diarrhoeal disease by age group.

We identified 54 studies that tested for viral pathogens. Rotavirus was the most common in the first three years of life, with peak prevalence in the third year. It was the most common source of diarrhoea in children aged >3 years (**Figure 2**, Table S1 in the **Online Supplementary Document**). Norovirus was the second most common, with peak prevalence in the first year of life and gradually declining to low levels by age three. Adenovirus and astrovirus were less commonly identified than rotavirus and norovirus, with the notable exception in the third year of life. Sapovirus was even less common, but only 13 studies that evaluated this pathogen included children under two.

**Figure 3:**
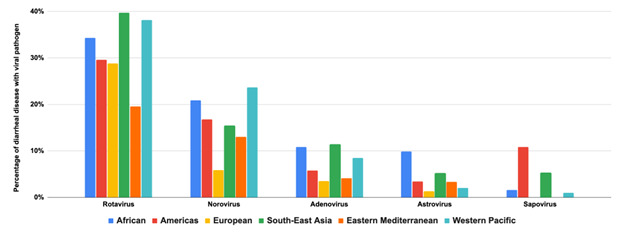
Viral pathogens are the cause of diarrhoeal disease by region.

Rotavirus was most common in children in SEAR, followed by WPR, AFR, AMR, EUR, and EMR, with a higher prevalence in the first three years of life (**Figure 3**). Norovirus and adenovirus occurred slightly more frequently in WPR and SEAR, respectively. Regarding the diarrhoeal aetiology studies focused on viruses across various geographic regions, rotavirus has the highest study count, with a total of 46 studies, predominantly in AFR with 14 cases. Adenovirus follows closely with 31 studies, while norovirus and astrovirus have 26 and 18 studies, respectively. Sapovirus shows the least representation, totalling only nine studies, with no cases reported in EUR. Overall, 135 studies were conducted across all regions (Table S3 in the **Online Supplementary Document**).

### Bacterial aetiologies among children aged <5 years with diarrhoea

**Figure 4:**
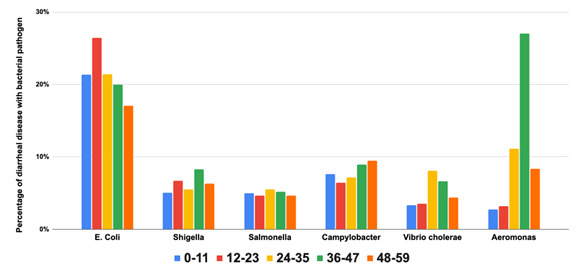
Bacterial pathogens are the cause of diarrhoeal disease by age group.

54 studies tested for bacterial pathogens. Toxigenic strains of *Escherichia coli* were common across all age ranges (**Figure 4**). *Aeromonas* was the second most identified pathogen, especially among children aged three to five years. *Campylobacter* was the next most common pathogen, with a prevalence ranging from 6–9% with a relatively even spread across age ranges. *Salmonella* and *Shigella* occurred in about 5% of children, with a relatively even spread across age ranges. *Vibrio cholerae* was also relatively evenly distributed across age ranges, although it was slightly more common in children aged >3 years.

**Figure 5:**
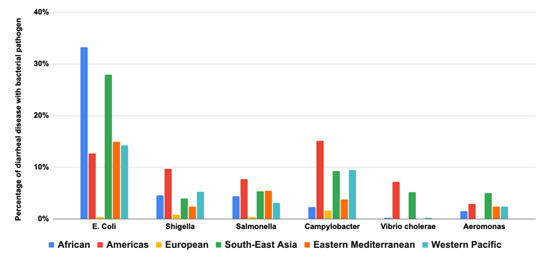
Bacterial pathogens are the cause of diarrhoeal disease by region.

The evidence shows clear regional differences in the distribution of some bacterial pathogens, with *Escherichia coli* identified most commonly in children in AFR and SEAR. In contrast, *Shigella* and *Campylobacter* were most common in AMR. *Vibrio cholerae* was mainly seen in children in the AMR and SEAR (**Figure 5**). Regarding the distribution of diarrhoeal aetiology studies focused on bacteria across different geographic regions, *Escherichia coli* leads with a total of 51 studies, primarily in Africa (AFR) with 16 cases. *Shigella* species closely follows with 47 studies, also showing significant representation in AFR. *Salmonella* species accounts for 39 studies, while *Campylobacter* species has 30 studies. *Vibrio* species and *Aeromonas* species have lower counts, with 17 and nine studies, respectively. The total number of studies conducted across all regions is 193 (Table S4 in the **Online Supplementary Document**).

### Parasitic aetiologies among children aged <5 years with diarrhoea

**Figure 6:**
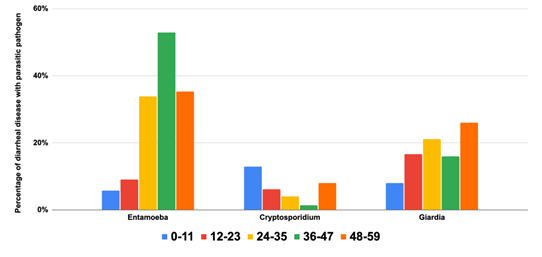
Parasitic pathogens are the cause of diarrhoeal disease by region.

31 studies tested for parasitic pathogens. The prevalence of *Entamoeba* species and *Giardia* species appeared to increase with age, with a higher prevalence among children aged three to five years (**Figure 6**). *Cryptosporidium* was less common but appeared to afflict more children in the first two years of life. Five studies used diagnostic tests to differentiate between species of *Entamoeba*. Notably, only two of the five papers on this protozoan used laboratory techniques such as quantitative reverse transcription polymerase chain reaction (PCR) and enzyme-linked immunosorbent assay [16,15].

**Figure 7:**
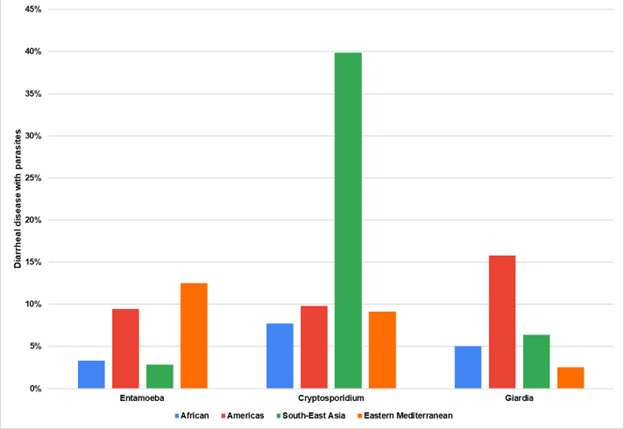
Parasitic pathogens are the cause of diarrhoeal disease by age group.

Parasites were distributed equally across global regions, except for a high percentage of children in the EMR suffering from *Entamoeba* species (**Figure 7**). Regarding the distribution of diarrhoeal aetiology studies focused on parasites across various geographic regions, *Entamoeba* species and *Cryptosporidium* species each have 20 studies, with significant representation in AFR. *Giardia* species follows closely with 21 studies, also prominent in AFR. Notably, no studies have been reported in EUR. Overall, the total number of studies conducted across all regions is 61 (Table S5 in the **Online Supplementary Document**).

### Aetiology among children aged six to 10 years

Only seven studies evaluated the aetiology of diarrhoea for children aged six to nine years. A 1991 study conducted in Thailand detected enteric viruses in 15% of the 156 samples [17]. Notably, rotavirus was identified in 13%, and astrovirus in 3% of children in this age range [17].

In a study conducted in Uttarakhand, India, analysis of isolated diarrhoeagenic bacteria revealed that *Escherichia coli* was the most prevalent bacteria (44.2%), followed by *Shigella*, *Salmonella*, *Klebsiella*, and *Campylobacter* (28.2%, 13.6%, 7.8%, and 6.1% respectively). Diarrhoea associated with all pathogens except *Klebsiella* primarily affected patients aged one to three years. *Klebsiella* predominantly affected infants below six months [18]. Another study conducted in Thailand between 2006–07 reported the highest cases of rotavirus and sapovirus in older children aged six to 10 years [19].

A study conducted in Kenya reported that sapovirus, astrovirus, and norovirus were relatively uncommon in children aged >5 years [20]. A study of older children and adults with acute diarrhoea in Shanghai, conducted between 2006–11, isolated eight pathogens – *Salmonella*, *Shigella*, *Vibrio cholerae*, *Vibrio parahaemolyticus*, EPEC, ETEC, EIEC, and EHEC [20]. A study conducted in Ethiopia in 2013 identified rotavirus and sapovirus infections in children aged >5 years [22]. In 2014, a study in Lebanon found seven cases of *Entamoeba histolytica*, two cases of rotavirus, and one case of adenovirus in children aged >5 years [23].

### Case-control analysis

In this section, we delve into key findings from papers that employed case-control methodology across various WHO regions. The inclusion of this separate analysis allows for a more detailed examination of specific study designs and their implications for understanding diarrhoeal disease patterns globally.

#### African Region

A 2017 study in Ethiopia focused primarily on protozoa and found that *Entamoeba* species and *Giardia lamblia* were the most common parasitic aetiologies of diarrhoea in children aged <5 years. However, some children also had infections due to *Salmonella* and *Shigella* species [24]. A study published in 2015 in rural Mozambique among children aged <5 years revealed that rotavirus was most common overall, while the highest number of diarrhoeal cases were caused by EAggEC among children between one to two years, and *Giardia lamblia* was responsible for a substantial proportion of cases among children between two to five years [13]. Lastly, a matched case-control study in 2016 in the Central African Republic also showed that rotavirus was the most prevalent virus causing diarrhoea among children below two years of age [15]. Notably, *Escherichia coli* and *Shigella* species have the highest study counts in the AFR, with 16 and 13 studies, respectively. A total of 120 studies were conducted in AFR, contrasting with only 10 in EUR (Table S2 in the **Online Supplementary Document**).

#### South-East Asian Region

A 2006 study in Mirzapur, Bangladesh, found that *Escherichia coli* was a major aetiology in age groups zero to one year and one to two years [9].

In Western Thailand, a case-control study in 2010 found that children aged <5 years were commonly affected by EPEC and ETEC [11]. Rotavirus, *Shigella*, adenovirus, and ETEC were significantly associated with acute diarrhoeal disease in children aged zero to one year old [11]. The major pathogens among children one to two years were rotavirus, adenovirus, *Shigella*, *and Giardia* [11]. *Shigella* had more cases for the age group two to three years compared to other pathogens, and it recorded the highest number of diarrhoeal cases in children aged >2 years [11].

In a case-control study published in 1999 in Dhaka, Bangladesh, rotavirus was the most frequent cause of diarrhoea in children aged <2 years in 1999, while *Campylobacter jejuni*, ETEC, *Aeromonas* species, and *Shigella* species were common in children aged two to three years [12]. Children aged three to four years were most affected by *Aeromonas* species, while *Campylobacter jejuni* was predominant in children aged four to five years [12]. Rotavirus and norovirus are also prevalent pathogens, particularly in the SEAR (Table S2 in the Online Supplementary Document).

#### Western Pacific Region

In China, a matched case-control study conducted in 2016 revealed that rotavirus was the predominant cause of diarrhoea in children aged <2 years, whereas *Shigella* was more prevalent among children aged two to five years [10]. In 2006, an aetiological study in Hanoi, Vietnam, revealed that group A rotavirus caused the highest incidence of diarrhoea [14].

Likewise, in 2002, a case-control study conducted in Hanoi, Vietnam, found group A rotavirus to be the leading pathogen with the highest number of cases in every age group from zero to five years [14]. Rotavirus was also prevalent in the WPR, consistent with the case-control study in Hanoi, Vietnam [14].

#### Global Enteric Multicentre Study

GEMS, conducted between 2007–11, included 9439 cases and 13 129 controls [6]. This multi-country analysis measured the population-based burden, aetiology, and clinical consequences of diarrhoea based on age, site, and pathogen, particularly in seven low-income countries in sub-Saharan Africa and South Asia, instead of focusing on all the six WHO regions [6]. The GEMS found five major pathogens responsible for causing diarrhoea in children aged <5 years, including rotavirus, *Cryptosporidium*, heat-stable toxin ETEC, typical EPEC, and *Shigella* (**Table 3**). More specifically, heat-stable toxin ETEC and typical EPEC were the most important pathogens in infants, whereas *Cryptosporidium* was the most important in ages one to two years. The attributable fraction of rotavirus was high at every site during infancy, although its prevalence diminished with age [6]. For bacterial pathogens, *Shigella* was associated with moderate-to-severe diarrhoea in infected children aged <5 years in all seven GEMS countries. The adjusted attributable fraction of *Shigella* progressed from infants to toddlers (one to two years). *Cryptosporidium* species were responsible for a higher incidence of episodes of moderate-to-severe diarrhoea in infants in all seven countries. Among parasites, *Cryptosporidium* species had the highest attributable fraction for infants in five countries and was associated with the highest number of deaths in toddlers between the ages of one and two years after EAggEC.

**Table 3:** Burden and aetiology of diarrhoeal disease in infants and young children from the Global Enteric Multicentre Study (GEMS) [6]

		Age in years (case/controls)		
**Location**	**Pathogens**	**0–1**	**1–2**	**2–5**
Basse, Gambia	Rotavirus, *Cryptosporidium*, ST-ETEC, *Shigella*, norovirus GII, adenovirus 40/41	400/585	455/639	174/345
Bamako, Mali	Rotavirus, *Cryptosporidium*, ST-ETEC, adenovirus 40/41, *Shigella*, *Entamoeba histolytica*	727/727	682/695	624/642
Manhica, Mozambique	Rotavirus, *Cryptosporidium*, adenovirus 40/41, ST-ETEC, *Shigella, Vibrio cholerae* O1	374/697	195/391	112/208
Nyanza Province, Kenya	Rotavirus, *Cryptosporidium*, ST-ETEC, *Shigella,* typical EPEC, non-typhoidal, *Salmonella*	673/673	410/621	393/589
Kolkata, India	Rotavirus, *Cryptosporidium*, ST-ETEC, *Shigella*, norovirus GII, adenovirus 40/41, *Vibrio cholerae* O1, *Campylobacter jejuni*, sapovirus	672/685	588/598	308/731
Mirzapur, Bangladesh	Rotavirus, *Cryptosporidium*, ST-ETEC, Shigella, *Aeromonas*, adenovirus 40/41, *Campylobacter jejuni*, Non-typhoidal *Salmonella*, *Entamoeba histolytica*, *Vibrio cholerae* O1, EAEC	550/878	476/761	368/826
Karachi, Pakistan	Rotavirus, *Cryptosporidium*, ST-ETEC, *Shigella*, *Aeromonas*, adenovirus 40/41, *Campylobacter jejuni*, *Entamoeba histolytica*, *Vibrio cholerae* O1	633/633	399/676	226/529

EAEC – enteroaggregative *Escherichia coli*, EPEC – enteropathogenic *Escherichia coli*, ST-ETEC – heat-stable enterotoxigenic *Escherichia coli*

#### Malnutrition and Enteric Disease Study

The important role of *Campylobacter* was highlighted in the Malnutrition and Enteric Disease Study (MAL-ED), a prospective cohort study conducted in multiple sites in South Asia, sub-Saharan Africa, and South America [25]. According to the secondary MAL-ED, which used non-diarrhoea stool samples from children, *Campylobacter* was responsible for declining linear growth in children aged <24 months in low-resource settings. Children in the second half of the first year of life were more vulnerable to *Campylobacter-*associated linear growth falls [26]. This conclusion is based on the consistency of the findings across various settings, the adjustment for confounding variables, the high incidence of infection, and the presence of a plausible mechanism. The study results concluded that promoting exclusive breastfeeding, routine drinking water treatment, targeted antibiotic administration, and access to improved latrines can decrease the burden of *Campylobacter* infections in these settings [26].

Another MAL-ED paper studied sapovirus in children aged <2 years in eight countries [27]. It is important to include this newer pathogen in rather than for future etiological studies and the use of culture-independent methods (e.g. multiplex PCR) to assess diarrhoeal aetiology. The pathogen was detected by real-time PCR. The incidence of sapovirus was highest in Peru and Bangladesh (with up to eight or more episodes per child-month of observation in these two countries vs less than 0.5 episodes per child-month in Brazil and South Africa). There was a higher burden of sapovirus from zero to 14 months of age, and then there was a decline in sapovirus cases. Enteric infection due to sapovirus had an increased odds of co-infection with rotavirus, astrovirus, adenovirus, and *Shigella*. A more recent longitudinal prospective study of sapovirus in Brisbane, Australia, further demonstrates the importance of this newer aetiology of diarrhoea [28]. The overall infection rate was much lower than Peru or Bangladesh in the MAL-ED (0.89 episodes per child-year), but the age range of peak infection rates was similar (six to 17 months). The lack of regular inclusion of sapovirus in diagnostic assessments has led to a limited understanding of its epidemiology and natural progression [26].

## DISCUSSION

Each year, 525 000 children die from diarrhoea, and 1.7 billion children deal with the repercussions to their health and well-being due to this disease [4]. We conducted a systematic review to identify the major aetiologies of diarrhoea in children in LMICs. The burden of moderate to severe diarrhoea can be greatly reduced by targeting five pathogens (rotavirus, *Shigella*, heat-stable toxin ETEC, *Cryptosporidium* species, and typical EPEC). Currently available interventions include rotavirus and cholera vaccines for prevention, antibiotics for the treatment of shigellosis, and oral rehydration and zinc for the management of all forms of acute diarrhoea [6].

In our systematic review, we revealed that most causes of childhood non-bloody acute diarrhoea are likely viruses or toxigenic strains of *Escherichia coli*. Overall, aetiologies such as shigellosis associated with invasive or bloody diarrhoea were less common. The predominance of viral and toxigenic *Escherichia coli* aetiologies in childhood non-bloody acute diarrhoea underscores a shift from invasive or bloody diarrhoea. This shift has practical implications, as most cases can be effectively managed with low osmolarity oral rehydration solution and zinc supplements, contributing to reduced severity and duration of diarrhoeal episodes [29,30]. The decline in shigellosis-associated diarrhoea further supports the evolving landscape of childhood diarrhoeal diseases. These findings have implications for managing childhood diarrhoea, as most children with acute diarrhoea can be managed with reduced (low) osmolarity oral rehydration solution and zinc supplements, which have been shown to reduce the severity and duration of diarrhoeal episodes [29,30].

While there were several important regional differences in aetiology, rotavirus was a major cause of diarrhoeal disease in children across all regions, especially in the first two years of life. This is consistent with the GEMS findings and is important since this is the only common aetiology of diarrhoea for which there is an effective vaccine [6]. Regional differences in aetiology highlight the complex epidemiology of childhood diarrhoea. Rotavirus emerges as a consistent major cause across diverse regions, emphasising its universal impact, especially in the first two years of life. The alignment with GEMS findings reinforces the significance of rotavirus vaccination globally [6]. These regional variations underscore the need for context-specific interventions while recognising universal challenges.

Important differences exist across studies regarding the breadth of pathogens they evaluated, sample size, and population studied. Some used classical microbiology (bacteriology), whereas, in more recent years, there has been greater use of culture-independent diagnostic strategies, largely based on PCR. In contrast to earlier studies of viral aetiology, which often used enzyme-linked immunosorbent assay for diagnosis, real-time PCR is much more widely used today, as exemplified by a recent case-control study of norovirus in children aged three months to five years conducted in Nepal [31].

Regional differences in aetiology highlight the complex epidemiology of childhood diarrhoea. Rotavirus emerges as a consistent major cause across diverse regions, emphasising its universal impact, especially in the first two years of life. The alignment with GEMS findings reinforces the significance of rotavirus vaccination globally [6]. These regional variations underscore the need for context-specific interventions while recognising universal challenges. Due to these methodological differences as well as differences in population characteristics and sampling strategies, we were unable to conduct a meta-analysis using the data from this systematic review.

In the eight case-control studies that were evaluated, *Vibrio cholerae*, *Escherichia coli*, *Aeromonas, Shigella* species, and rotavirus were more frequently encountered in cases than controls. Some studies showed that pathogens such as adenovirus, norovirus, and *Salmonella* had more cases than controls. However, a few studies recorded more controls than cases for these pathogens. *Giardia* recorded more controls than cases in seven case-control studies, excluding the GEMS paper, which did not report *Giardia* in its pathogens. Consequently, interpretation of studies without control populations, especially when they are assessing giardiasis, salmonellosis, and certain viral pathogens, need to be cautiously interpreted as these pathogens may be colonising the gastrointestinal tract rather than causing acute illness.

Our study also emphasises the interconnectedness of environmental factors and diarrhoeal disease transmission. Climate change amplifies waterborne disease risks, affecting transmission pathways for pathogens like *Vibrio cholerae*, *Cryptosporidium*, and *Leptospira* species through rising temperatures and extreme weather events. Vulnerable populations with insufficient infrastructure face an increased incidence of waterborne diseases, underscoring the urgency of global climate action and enhanced public health measures [32].

### Limitations

There are several limitations of the published literature on diarrhoeal aetiology that are important to acknowledge. The studies included in the review spanned from 1990 to 2020. The analysis did not consider the temporal aspect of the data, potentially affecting the relevance of the reported diarrhoeal disease aetiology. A bias assessment of the aetiology results was not performed, given the broad variability in study design, microbiological methods, and populations studied. Additionally, the narrow focus on specific pathogens and limited detection efforts, especially for pathogens like sapovirus, may result in an incomplete understanding of diarrhoeal disease aetiology. Furthermore, the choice of study populations could introduce selection bias, possibly overrepresenting certain regions or populations and impacting the generalizability of the findings. The pooling of *Entamoeba histolytica* and *Entamoeba dispar* into one category of *Entamoeba* species was a limitation as this mixed pathogenic with non-pathogenic protozoal species. Likewise, stool microscopy is ineffective because the morphology is identical and thus does not allow for distinguishing between *Entamoeba dispar* and *Entamoeba histolytica*. The enzyme-linked immunosorbent assay for antigen and PCR are the two acceptable laboratory methods that allow for differentiation between the two species, the non-pathogenic *Entamoeba dispar* and pathogenic *Entamoeba histolytica*.

Our review found important differences in the microbiological methodologies used to identify diarrhoea-causing pathogens. As noted above, culture-independent methods (e.g. multiplex PCR) have become widely used. Even though they are highly sensitive and may thus detect intestinal colonisation and actual disease, they can be used to improve our understanding of diarrhoea aetiology. The regional variability is complex and limited by the number of studies and the data quality collected. In designing this systematic review, we excluded studies that focused on single pathogens. While this decision led to the exclusion of many single aetiology studies, we believe that this was appropriate since the inclusion of these studies would have skewed the results towards the pathogen described in these studies.

Many were single-site studies of just a few pathogens, a narrow age range, and a highly selected population. Thus, they are not likely to represent the overall situation within a country or a WHO region. Similarly, only 38 out of the 75 studies evaluated a full year of diarrhoeal episodes to assess the seasonality of aetiology. A recent study in 2023 in Guinea Bissau by Kantele et al. demonstrated the importance of how seasonality influences the distribution of stool pathogens, thus emphasising the importance of exploring the relationship between seasonality and the aetiology of diarrhoea among children [27].

Another major challenge was the lack of data for children between six and 10 years old. While children under five have been studied thoroughly, older children have been neglected in aetiology studies, although the burden of disease in this older child population has not been well characterised. In addition, most articles that were searched for inclusion did not include stratification by sex for each pathogen and age group. This further limitation should be considered for future research in this area. This systematic review also did not assess bias when evaluating the studies as critical appraisal tools to evaluate the quality of the papers were not included in the review process. Another limitation of our study is that many studies did not use microbiological methods that would allow differentiation of pathogen *Entamoeba histolytica* from non-pathogenic *Entamoeba dispar.*

### Future studies

The scarcity of data for older children (six to 10 years), coupled with the absence of sex stratification in many studies, emphasises the need for future research to address these gaps. Critical appraisal tools could enhance the evaluation of study quality. Considering seasonality's impact on pathogen distribution, future research should explore its relationship with diarrhoea aetiology among children [27]. Ideally, future studies should utilise multi-site collaborations using case-control designs to understand the causes of diarrhoeal in children older than five. In addition, a longitudinal study of broad age groups focusing on young children and adolescents for a minimum of one year would help understand the differences in data according to WHO regions, pathogens, and seasons. A study of this kind might also compare the aetiology of diarrhoea in urban and rural communities. In the future, the impact of extreme weather conditions and global warming on diarrhoeal disease risk in children in LMICs will be increasingly important to assess.

New research should also utilise culture-independent methods (e.g. multiplex PCR) to assess diarrhoea aetiology and include sapovirus among the viral diarrhoea aetiologies being evaluated. Understanding the aetiology of childhood diarrhoea can enhance our knowledge of the primary pathogens, paving the way for the development of vaccines aimed at preventing morbidity from diarrhoeal diseases in young children in the future. Similarly, better evidence on aetiology can help limit antibiotic use to pathogens most likely to be associated with invasive diseases and thereby help reduce the global burden of antibiotic resistance.

## CONCLUSIONS

In conclusion, in this systematic review, we aimed to identify the predominant pathogens associated with diarrhoeal disease across different age groups and regions. However, it is essential to acknowledge certain limitations, such as the lack of consideration for the temporal aspect of the data, the narrow focus on specific pathogens, and potential selection bias.

To ensure a more comprehensive understanding of diarrhoeal disease aetiology and to enable targeted prevention and control strategies for improved public health outcomes, we must address these limitations through future research. It is crucial to scale up global access to the rotavirus vaccine, given the significant disease burden caused by the pathogen [6].

Let us act by adapting global policies for prevention and treatment, considering local contexts and scientific evidence to ensure their effectiveness. Urgently needed interventions include improving sanitation and hygiene and promoting novel vaccines for enteric pathogens beyond rotavirus. To achieve these goals, collaboration among international organisations, governments, and local communities is necessary. Together, we can implement these policies and work towards better health outcomes for all.

## Additional material


Online Supplementary Document
