# Supplementary material for: Aetiology of diarrhoea in children aged zero to nine years in low- and middle-income countries: A systematic review
Source: J Glob Health. 2024 Nov 1;14:04168. doi: 10.7189/jogh.14.04168 (PMC11529147; doi:10.7189/jogh.14.04168)
Supplement: Online Supplementary Document [file jogh-14-04168-s001.zip › jogh-14-04168-s001.pdf]

**Table S1:** The number of diarrhoeal aetiology studies by pathogen and age group (cases)

| | | Age group (months) | | | | |
|---|---|---|---|---|---|---|
| **Pathogens** | **Organism type** | **0-11** | **12-23** | **24-35** | **36-47** | **48-59** |
| Rotavirus | Virus | 43 | 33 | 12 | 8 | 8 |
| Norovirus | Virus | 25 | 21 | 5 | 2 | 2 |
| Adenovirus | Virus | 29 | 23 | 8 | 1 | 1 |
| Astrovirus | Virus | 18 | 15 | 4 | 2 | 1 |
| Sapovirus | Virus | 8 | 7 | 0 | 0 | 0 |
| *Escherichia coli* | Bacteria | 47 | 36 | 13 | 10 | 9 |
| *Shigella* | Bacteria | 39 | 24 | 7 | 10 | 10 |
| *Salmonella* | Bacteria | 33 | 21 | 5 | 6 | 6 |
| *Campylobacter* | Bacteria | 29 | 18 | 7 | 5 | 5 |
| *Vibrio cholerae* | Bacteria | 11 | 6 | 2 | 3 | 2 |
| *Aeromonas* | Bacteria | 8 | 4 | 3 | 3 | 2 |
| Entamoeba | Parasite | 14 | 10 | 3 | 5 | 5 |
| *Cryptosporidium* | Parasite | 18 | 14 | 5 | 2 | 2 |
| *Giardia* | Parasite | 17 | 13 | 5 | 3 | 5 |

| | | | | | | |
|---|---|---|---|---|---|---|
| Total = 294 | | | | | | |

**Table S2:** The number of diarrhoeal aetiology studies by pathogen and geographic region (cases).

| **Pathogens** | **AFR** | **AMR** | **EMR** | **EUR** | **SEAR** | **WPR** |
|---|---|---|---|---|---|---|
| Rotavirus | 14 | 9 | 6 | 2 | 9 | 6 |
| Norovirus | 10 | 4 | 1 | 1 | 6 | 4 |
| Adenovirus | 9 | 7 | 2 | 2 | 7 | 4 |
| Astrovirus | 6 | 4 | 1 | 1 | 3 | 3 |
| Sapovirus | 4 | 1 | 0 | 0 | 2 | 2 |
| *Escherichia coli* | 16 | 7 | 8 | 1 | 8 | 11 |
| *Shigella* spp. | 13 | 7 | 7 | 1 | 11 | 8 |
| *Salmonella* spp. | 12 | 5 | 5 | 1 | 10 | 6 |
| *Campylobacter* | 7 | 5 | 4 | 1 | 9 | 4 |
| *Vibrio* spp. | 4 | 2 | 0 | 0 | 7 | 4 |
| *Aeromonas* spp. | 3 | 1 | 1 | 0 | 2 | 2 |
| Entamoeba spp. | 8 | 5 | 4 | 0 | 3 | 0 |
| *Cryptosporidium* spp | 8 | 4 | 4 | 0 | 3 | 1 |

| | | | | | | |
|---|---|---|---|---|---|---|
| *Giardia* spp. | 6 | 5 | 4 | 0 | 5 | 0 |
| Total | 120 | 66 | 47 | 10 | 85 | 55 |

**Table S3:** The number of diarrhoeal aetiology studies by viruses and geographic region.

| **Pathogens** | **AFR** | **AMR** | **EMR** | **EUR** | **SEAR** | **WPR** | **Total** |
|---|---|---|---|---|---|---|---|
| Rotavirus | 14 | 9 | 6 | 2 | 9 | 6 | 46 |
| Norovirus | 10 | 4 | 1 | 1 | 6 | 4 | 26 |
| Adenovirus | 9 | 7 | 2 | 2 | 7 | 4 | 31 |
| Astrovirus | 6 | 4 | 1 | 1 | 3 | 3 | 18 |
| Sapovirus | 4 | 1 | 0 | 0 | 2 | 2 | 9 |
| Total | 48 | 25 | 10 | 6 | 27 | 19 | 135 |

**Table S4:** The number of diarrhoeal aetiology studies by bacteria and geographic region.

| **Pathogens** | **AFR** | **AMR** | **EMR** | **EUR** | **SEAR** | **WPR** | **Total** |
|---|---|---|---|---|---|---|---|
| *Escherichia coli* | 16 | 7 | 8 | 1 | 8 | 11 | 51 |
| *Shigella* spp. | 13 | 7 | 7 | 1 | 11 | 8 | 47 |
| *Salmonella* spp. | 12 | 5 | 5 | 1 | 10 | 6 | 39 |
| *Campylobacter* | 7 | 5 | 4 | 1 | 9 | 4 | 30 |
| *Vibrio* spp. | 4 | 2 | 0 | 0 | 7 | 4 | 17 |
| *Aeromonas* spp. | 3 | 1 | 1 | 0 | 2 | 2 | 9 |
| Total | 55 | 27 | 25 | 4 | 47 | 35 | 193 |

**Table S5:** The number of diarrhoeal aetiology studies by parasites and geographic region.

| **Pathogens** | **AFR** | **AMR** | **EMR** | **EUR** | **SEAR** | **WPR** | **Total** |
|---|---|---|---|---|---|---|---|
| *Entamoeba* spp. | 8 | 5 | 4 | 0 | 3 | 0 | 20 |
| *Cryptosporidium* spp. | 8 | 4 | 4 | 0 | 3 | 1 | 20 |
| *Giardia* spp. | 6 | 5 | 4 | 0 | 5 | 1 | 21 |
| Total | 22 | 14 | 12 | 0 | 11 | 2 | 61 |
